# Data sources on COVID-19 infection and vaccination in pregnancy on the island of Ireland: strengths, weaknesses, and recommendations for future pandemic preparedness

**DOI:** 10.12688/hrbopenres.14011.2

**Published:** 2025-03-07

**Authors:** Melissa Kelly, Joanne Given, Julie Arnott, Helen Dolk, Richard A. Greene, Ali S. Khashan, Seamus Leonard, Mairéad Madigan, Mary T. O’Mahony, Maria Loane, Gillian M. Maher

**Affiliations:** 1 INFANT Research Centre, University College Cork School of Public Health, Cork, County Cork, T12 K8AF, Ireland; 2 Ulster University Institute of Nursing and Health Research, Belfast, Northern Ireland, BT15 1ED, UK; 3 University College Cork School of Public Health, Cork, County Cork, T12 K8AF, Ireland; 4 School of Medicine, Ulster University, Londonderry, Northern Ireland, BT48 7JL, UK; 5 National Perinatal Epidemiology Centre, University College Cork, Cork, County Cork, T12 K8AF, Ireland; 6 Department of Obstetrics and Gynaecology, Cork University Maternity Hospital, Cork, Co. Cork, T12 YE02, Ireland; 7 HSE National Immunisation Office, Dublin, D08 XW6R, Ireland; 8 Health Protection Surveillance Centre, Dublin, D01 A4A3, Ireland; 9 Department of Public Health HSE-South, St Finbarr's Hospital, Cork, T12 XH60, Ireland

**Keywords:** COVID-19 infection, Maternal and child health, Pregnancy, Administrative Health Data, COVID-19 Vaccines

## Abstract

**Background:**

Monitoring coronavirus disease (COVID-19) infection and vaccination during pregnancy is vital because of the increased susceptibility to severe disease. This article outlines the available data sources on COVID-19 infection and vaccination rates during pregnancy in Northern Ireland (NI) and the Republic of Ireland (ROI) and describes the processes, strengths, and weaknesses of available data.

**Methods:**

Three data sources on COVID-19 vaccination and infection were identified in the ROI: the national computerized infectious disease reporting (CIDR) system used for reporting notifiable infectious diseases, the national dataset of all COVID-19 vaccinations for all residents (COVAX), and a regional Maternal and Newborn Clinical Management System (MN-CMS), which includes data on COVID-19 vaccination and infection. Four data sources were identified in NI: the NI maternity system (NIMATS) records maternity data, including COVID-19 infection and vaccination during pregnancy; datasets of COVID-19 antigen testing performed in hospitals (Pillar 1) and in the community (Pillar 2); and the NI Vaccine Management System dataset of COVID-19 Vaccinations.

**Results:**

In the ROI, the CIDR database allows for the calculation of COVID-19 infection rates in women of reproductive age; however, pregnancy status remains largely unreported. The COVAX dataset includes pregnancy status, although the accuracy depends on whether the pregnancy is known at the time of vaccination. The MN-CMS tracks COVID-19 infection and vaccination during pregnancy. However, there are uncertainties regarding its reliability. In NI, COVID-19 data are available for all pregnant women using Health and Care numbers to link the NIMATS data to testing and vaccination databases.

**Conclusions:**

Both countries track COVID-19 infection and vaccination rates, but the strength of the NI system is the use of unique identification numbers that allow linkage of maternal records to infection and vaccination databases. Both countries face delays in data access, underscoring the need for real-time systems to support future pandemic preparedness.

## Background

On the 11th of March 2020, the World Health Organization (WHO) declared SARS-CoV-2 (COVID-19) as a global pandemic ^1^. Emerging evidence has demonstrated the adverse effects of COVID-19 on vulnerable populations, particularly on pregnant women ^2^. The impact of COVID-19 on pregnant women and their unborn babies is multifaceted because of the potential risks associated with the virus itself, medicines to treat the virus, safety of vaccines, and indirect effects of pandemic-related stress ^3^. COVID-19 infection during pregnancy may negatively impact maternal health and fetal development and increase the risk of preterm birth and congenital anomalies (CA; 3, 4). Despite the potential risk of infection, a high proportion of pregnant women remain unvaccinated during the pandemic, largely because of vaccine hesitancy ^5^. Longitudinal follow-up studies using population-based datasets of women vaccinated earlier in pregnancy are required to assess maternal, pregnancy, and infant outcomes. However, the availability of detailed, accurate, and timely data is crucial to achieve this.

On the Island of Ireland (IOI), pregnancy-related data were collected through clinical and administrative processes within healthcare settings during the pandemic. The IOI comprises the Republic of Ireland (ROI), a sovereign country within the European Union (EU), and Northern Ireland (NI), which is part of the United Kingdom (UK). The national datasets in the ROI ( https://covid19ireland-geohive.hub.arcgis.com/) and NI ( https://www.health-ni.gov.uk/articles/covid-19-dashboard-updates) provided critical information and reported confirmed cases of COVID-19 infection by age, sex, and other demographic variables. However, there is a significant lack of data specific to pregnant women. While extensive research on COVID-19 has been conducted using administrative data from the IOI ^6, 7^, studies specifically addressing the impact of the virus during pregnancy are lacking. This deficit in the understanding of the impact of COVID-19 during pregnancy highlights the urgent need for more comprehensive data collection to ensure future pandemic preparedness.

This article aimed to describe the strengths and weaknesses of available data sources on COVID-19 infection and vaccination uptake rates during pregnancy on the IOI, with differing healthcare systems in the two jurisdictions. Based on our findings, we suggest recommendations for improving access to data for research on pandemic preparedness during pregnancy.

## Methods

### Available data sources

In the ROI, population-based data on COVID-19 infection and vaccination were managed by the Irish government health system’s Health Protection Surveillance Centre (HPSC), which is part of the broader Health Service Executive (HSE; https://www.hse.ie/). In the NI, COVID-19 data were managed using the Health and Social Care (HSC) system ( https://www.health-ni.gov.uk/). Eligible data sources included individual-level information on COVID-19 infection or vaccination status in conjunction with or linkable to pregnancy status.

The data sources were identified by a research team in April 2023. Seven potential datasets containing data on COVID-19 infection and vaccination during pregnancy in the IOI were identified ( Table 1). In the ROI, three data sources on COVID-19 infection and vaccination were identified: the national computerized infectious disease reporting (CIDR) system used for reporting notifiable infectious diseases ^8^, a national dataset of all COVID-19 vaccinations (COVAX) for all residents ^9^, and a regional Maternal and Newborn Clinical Management System (MN-CMS), which includes data on COVID-19 infection and vaccination in four maternity hospitals ^10^. There is no information on the size of the datasets in ROI, but the MN-CMS dataset will cover all women and babies in the maternity services in the ROI when the electronic health records are developed. In NI, four data sources were identified: the national NI maternity system (NIMATS) which records information on approximately 25,000 births per year in NI and includes data on infection and vaccination rates during pregnancy; national datasets of COVID antigen tests performed in the hospital (Pillar1) and community (Pillar 2) ^11, 12^; and the national COVID-19 Vaccination dataset extracted from the NI Vaccine Management System ^13^. The Pillar 1, Pillar 2, and vaccination datasets cover the whole population of NI.

**Table 1.** Key components of COVID-19 infection and vaccination in pregnancy databases on the island of Ireland.

	Source	Data	First year data source established	Managed by	Geographical Coverage	Direct cost to access data
**ROI**	Computerised Infectious Disease Reporting (CIDR)	COVID-19 Infection	2004	Health Service Executive (HSE)	All of ROI	No
	COVAX	COVID-19 Vaccination	2021	HSE	All of ROI	No
	Maternal and Newborn Clinical Management System (MN-CMS)	Maternal and Newborn Electronic Health Record	2016	HSE	Cork University Maternity Hospital (CUMH), University Hospital Kerry (UHK), Rotunda Hospital, and National Maternity Hospital (NMH)	No
**NI**	COVID-19 Infection Database Pillar 1 (hospitals)	COVID-19 Infection	2020	Honest Broker Service (HBS)	All of NI	Yes
	COVID-19 Infection Database Pillar 2 (community)	COVID-19 Infection	2020	HBS	All of NI	Yes
	COVID-19 Vaccination Database	COVID-19 Vaccination	2021	HBS	All of NI	Yes
	Northern Ireland Maternity System (NIMATS)	Maternal and Newborn Electronic Health Record	2010	HBS	All of NI	Yes

***Availability of unique identification numbers***

In the ROI, Individual Health Identifiers (IHI) were successfully implemented to facilitate the COVID-19 vaccination program during the COVID-19 pandemic. To date, the IHI has not yet been fully implemented in all healthcare settings; however, progress is being made with the IHI being rolled out across the ROI healthcare system to enhance the linkage of medical records across different healthcare organizations ^14^. In NI, every individual has a unique Health and Care Number (HCN) that can be used by HSC to link the records of an individual across multiple data sources ^15^.

## Results

### Description and access of ROI data sources

***Computerised Infectious Disease Reporting (CIDR)***

The CIDR is an information system developed to report notifiable infectious diseases in the ROI for the surveillance and management of infectious diseases ^8^. During the pandemic, COVID-19 was added to the national list of notifiable diseases, and the CIDR was programmed to collect data on each COVID-19 infection as per the COVID-19 case definition and case form, which included pregnancy status at the time of infection. A multidisciplinary range of healthcare and public health professionals collected CIDR data throughout the COVID-19 pandemic, including public health nurses, doctors, and laboratory staff. When COVID-19 contact tracing centers were established to support the pandemic response by contact tracing each case of COVID-19 infection, the enhanced surveillance of COVID-19 cases was taken over by the positive patient assessment (PPA) centers conducted by the COVID-19 contact tracers in the ROI’s contact management program. The PPA contains the enhanced surveillance section of the COVID-19 case form, which includes questions designed to assess the patient’s lifestyle factors, medical background, and behavioral patterns. Data on confirmed COVID-19 cases accounted for over 99% of the cases reported by the CIDR. Due to demands during surge capacity, PPA data collection was limited to certain peaks, impacting the completeness of pregnancy status. From February 2022, the focus of surveillance shifted to severe cases of COVID-19 only, thereby reducing the number of PPAs conducted. In addition, from February 2022 onwards, data on pregnancy is less complete and therefore less reliable for COVID-19 cases from this point onwards.

**Data access:** Access to CIDR data is restricted and requires approval from the CIDR Peer Review Group following a thorough application review. A data access request from the outlining details of the proposed project was submitted to CIDR’s National Peer Review Coordinator ( cidrdatarequests@hpsc.ie). The CIDR Peer Review Group reviewed the application and requested further details, where necessary. Accessing the CIDR data took approximately 12 months, from the submission of the data request form to receiving the dataset for analysis.

***COVAX***

COVAX now serves as the HSE’s Vaccination Platform dedicated to managing, monitoring, and supporting the administration of COVID-19, Influenza (flu), and pneumococcal vaccinations throughout the ROI ^9^. COVAX was established to record all COVID-19 vaccinations administered to the public. A range of healthcare and administrative staff collected COVAX data including vaccination center staff, hospital staff, and pharmacists. This comprehensive electronic dataset also contained information on individuals who were not vaccinated due to non-attendance (i.e., those scheduled for vaccination but who did not attend), ensuring a thorough record of vaccination efforts across the ROI. Pregnancy status at the time of the vaccination schedule was also included in the COVAX data. The entire population was offered vaccination against COVID-19, according to the national defined priority, with those at the highest risk being vaccinated first. Residents of elderly care homes and healthcare workers were the first two groups to be vaccinated, whereas pregnant women were first offered the COVID-19 vaccine in May 2021. Vaccines administered in ROI throughout the COVID-19 pandemic included Pfizer, Astra Zeneca, Moderna, Novavax, and Janssen.

**Data access:** We submitted a COVAX Data Share Request form outlining the proposed project, including insurance details (liability), to Integrated Information Services (IIS) ( iis.team@hse.ie), the main data analytics service for the HSE. This was reviewed by the internal data governance team and referred to the HSE Data and Information Management Group (DAIM). The DAIM required a Reference Request form and a Privacy Impact Assessment form to be completed (which also included a meeting with the DAIM). Following approval from DAIM and the Data Protection Officer, each member of the research team completed a ShareFile Access Request form before accessing a project-specific folder on the IIS. Accessing the COVAX data took approximately 14 months.

***Maternal and Newborn Clinical Management System (MN-CMS)***

The MN-CMS has been rolled out in four of the 19 maternity units in the ROI and enables maternity data to be recorded on an electronic health record (EHR), allowing all maternal and newborn information to be stored in one record ^10^. Data were collected during routine delivery of maternity care, including, but not limited to, demographics, lifestyle factors, medical history, pregnancy factors such as any complications of pregnancy, delivery data, and data relating to the newborn by hospital staff, including midwives and obstetricians. During the COVID-19 pandemic, data related to infection and vaccination status were collected at the time of booking for expecting mothers. 

**Data access:** This study was approved by the Clinical Research Ethics Committee of Cork Teaching Hospitals for Cork University Maternity Hospital (CUMH). Following approval, a research application form was submitted to the Local Information Governance Group. A more detailed description of how to access MN-CMS data, including anticipated timelines and processes, has recently been published ^16^. The approximate timeline from the initial request to data access for MN-CMS data ranges from to 6–12 months, depending on the number of maternity units and the number of variables being requested. Upon review of the MN-CMS data, it was concluded that the information regarding COVID-19 infection and vaccination within this data source was inadequate, and there were uncertainties regarding its reliability. While the exact reasons for this are poorly understood, it is partly because pregnant women with COVID-19 likely avoided hospitals and there is no access to their GP data regarding infection and vaccination.

### Description and access of NI data sources

***Northern Ireland Regional Maternity system (NIMATS)***

NIMATS is a regional maternity care system that records demographic information and maternity care for pregnant individuals across all five Health Trusts in NI. This system captures data related to the current pregnancy and details concerning the mother’s medical and obstetric history. The NIMATS provides important data on various aspects of childbirth, including birth number, interventions, maternal risk factors, birth weight, maternal smoking, body mass index (BMI), and breastfeeding status upon discharge. NIMATS data are collected by hospital staff, including midwives, obstetricians, nurses, and GPs. In June 2020, data relating to infection at booking, delivery, and discharge and any admissions for COVID-19 infection during pregnancy and infant COVID status were added to NIMATS. In March 2021, the COVID-19 vaccination status, including the number and dates of vaccines, was added. These COVID infection and vaccination data have not yet been evaluated for research purposes.

***COVID-19 infection databases***

COVID-19 infection testing in the NI was organized into two primary pillars: pillar 1 and pillar 2. Pillar 1 testing focused on testing individuals hospitalized with COVID-19 symptoms, healthcare workers, and residents and staff of care homes. The objective of pillar 1 testing was to identify and manage COVID-19 cases in high-risk settings. Testing within pillar 1 is typically conducted using polymerase chain reaction (PCR) and data were collected by hospital staff, including nurses and doctors. Pregnant women are typically tested upon admission for delivery.

Pillar 2 testing was community-based and involved testing centers, mobile testing units, and home testing kits. Data were collected by testing center staff, and self-reported data from at home testing kits were uploaded onto the COVID-19 government dashboard. This pillar targeted individuals in the general population who experienced symptoms of COVID-19 or had been in close contact with confirmed cases. The primary goal of pillar 2 testing was to identify and isolate cases of COVID-19 within the community, thereby reducing the transmission of the virus and preventing outbreaks. Pillar 2 testing also uses PCR tests along with other testing methods, such as rapid antigen tests ^17^. In NI, Pillar 2 testing was managed by the Department of Health and the Public Health Agency, with testing sites and facilities strategically located across the region to ensure accessibility for the population. The positive results of rapid testing at home are included in the Pillar 2 database, but this voluntary notification decreased in the later stages of the pandemic. COVID-19 Infection Database (Pillar 2) data were collected by testing centre staff, self-reported data from at home testing kits.

Overall, the combination of pillar 1 and pillar 2 testing strategies in NI allowed for comprehensive testing coverage, targeting both high-risk settings and the broader community to effectively monitor and control the spread of COVID-19. Both Pillar 1 and Pillar 2 data sources included the date of the positive test, sex, age at time of test, date of birth, and patients’ HCN.

***COVID-19 Vaccination Database***

In NI, the objective of the COVID-19 Vaccine Programme was to vaccinate members of the population at the highest risk of serious illness or death. These data were collected by vaccination center staff, hospital staff, and pharmacists. Over time, vaccinations were offered to every member of the NI population aged over five years. This dataset includes details of the patient’s date of vaccination, sex, age at vaccination, date of birth, and the patient’s HCN. Vaccines administered in NI throughout the COVID-19 pandemic included Pfizer, Astra Zeneca, Moderna, and Novavax.

**Data Access:** For this study, access to COVID-19 infection, COVID-19 vaccination, and NIMATS databases were obtained via the HBS. The HBS provides approved researchers with access to linked, de-identified health data in a safe setting. The HBS requires institutional approval before the application is submitted to the HBS Governance Board. Patient and Public Involvement and Engagement are also required before application approval. The HBS charges £570 a day (including VAT) to create datasets with the final cost depending on the complexity of the dataset requested (time period covered and number of datasets linked). The approximate timeline from the initial request to data access for NI depends on the number of datasets and variables requested, and it took approximately 6–12 months for access to NIMATS linked to the COVID datasets.

Further descriptive details of each data source on COVID-19 infection and vaccination during pregnancy in the IOI were identified ( Table 2).

**Table 2.** Description of data sources on the island of Ireland.

	Data source	Availability of date of data collected	Relevant Variables Included	How often data were updated	Who was responsible for data collection	When and where data were collected	Average Length of Data Access	Application to access	Who can access this data	Metadata available
**ROI**	**CIDR**	March 2020 - present	Pregnancy status at the time of COVID-19 infection	Weekly	Public health nurses, doctors, and laboratory staff	During visits to the hospital, testing sites, and contact tracing centres	12 months	Submit data access request from the outlining details of the proposed project was submitted to CIDR’s National Peer Review Coordinator ( cidrdatarequests@ hpsc.ie)	Data requests from researchers will be assessed on a case-by- case basis.	Unavailable
	**COVAX**	Dec 2020 – present	Pregnancy status at the time of COVID-19 vaccination	Weekly	Vaccination center staff, hospital staff, and pharmacists	Upon visits to vaccination sites, pharmacies, GP clinics, hospitals, and other healthcare facilities	14 months	Submit a COVAX Data Share Request form outlining the proposed project, including insurance details (liability), to Integrated Information Services (IIS) ( iis.team@hse.ie), the main data analytics service for the HSE.	Data requests from researchers will be assessed on a case-by- case basis.	Unavailable
	**MNCMS**	2020- present	COVID-19 infection and vaccination status at the time of appointment booking for expecting mothers	When women visited the maternity unit	Hospital staff, including midwives, obstetricians	During maternity appointments in the hospital	6-12 months	A request should be made to the clinical lead within each maternity unit where data is being requested ( https://www. ehealthireland.ie/technology- and-transformation-functions/ acute-delivery/maternal- newborn-clinical-management- system-mn-cms/mn-cms- overview/)	Individuals affiliated with an education, healthcare or other research institution.	Unavailable
**NI**	**Pillar 1**	2020- present	Cases of COVID-19 Infection of individuals hospitalized with or without COVID-19 symptoms, healthcare workers, and residents and staff of care homes	Daily	Hospital staff including nurses and doctors	During hospital visit	6-12 months	Applications to Honest Broker Service at https://bso.hscni. net/directorates/digital/honest- broker-service/honest-broker- service-researcher-access/	Accredited Researchers; Department of Health; HSC Organisations which have signed up to the Memorandum of Understanding	https://bso. hscni.net/ directorates/ digital/honest- broker-service/ honest-broker- service- researcher- access/metadata/
	**Pillar 2**		Cases of COVID-19 Infection of individuals in the general population who experienced symptoms of COVID-19 or had been in close contact with confirmed cases	Daily	Testing centre staff, self- reported data from at home testing kits	Self-reported results of at home testing kits on the government COVID-19 dashboard and upon visit to testing centres and mobile testing units				
	**Vaccine**		Vaccination records of members of the public	Weekly	Vaccination center staff, hospital staff, and pharmacists	Upon visits to vaccination sites, pharmacies, GP clinics, hospitals, and other healthcare facilities				
	**NIMATS**	2010- present	COVID-19 infection throughout pregnancy and vaccination status at delivery	Daily	Hospital staff, including midwives and obstetricians.	During maternity appointments.				

## Discussion

This article describes the existing data sources available on COVID-19 infection and vaccination during pregnancy on the IOI as well as their strengths and weaknesses. Across the IOI, there were key differences in the data access procedures. In the ROI, each of the available datasets involved separate applications with various procedures. In NI, access was more streamlined and could be requested through a central data provider with the existence of an HCN to support data linkage. Conversely, while the implementation of IHI numbers in the ROI continues to be rolled out, with the COVID-19 pandemic arguably expediting this implementation, it has not yet been fully implemented in all health care settings.

Several notable differences between the two jurisdictions on the IOI emerged when requesting data on COVID-19 infection and vaccination during pregnancy. In the ROI, the process of data access appeared fragmented, marked by disparate systems and the inability to link pregnant women’s data throughout the healthcare system. Furthermore, applications for data access in the ROI often necessitate the addition of an HSE representative to the research team. Conversely, in NI, data access procedures demonstrated a more integrated approach with various components of the healthcare system linked by HCNs for more streamlined access via a single data provider, the HBS. These differences in accessing pregnancy-related COVID-19 data in the ROI and NI highlight the disparate infrastructure between the jurisdictions that affect research capabilities.

## Strengths, limitations and recommendations

To further facilitate research using available data on COVID-19 infection and vaccination during pregnancy, recommendations and limitations are discussed. First, the lack of an expected timeframe for accessing data in both the ROI and NI prevents researchers from effectively managing expectations and communicating timelines with stakeholders and funders. Researchers need to have detailed timeframes to gain access to these data and effectively plan their research projects. Knowing the duration of data access processes allows researchers to schedule their workloads effectively, set realistic timelines for project completion, and allocate appropriate resources. Data can quickly become outdated, and an upper time limit on data access requests would ensure that information used in research is timely enough to inform recommendations for current public health practices. Furthermore, many data protection and privacy regulations, such as the General Data Protection Regulation (GDPR), impose specific time constraints on data usage and retention.

Accessing and analyzing available data on COVID-19 infection and vaccination during pregnancy is crucial for evaluating public health guidelines and improving research on maternal and child health across the IOI. In NI, there are clear processes for requesting and accessing data on COVID-19 infection and vaccination during pregnancy, while the arrival of the European Health Data Space (EHDS) places a requirement on the ROI to facilitate access to health data for researchers ^18^. Having detailed instructions for accessing population-based and maternal data on COVID-19 infection, vaccination, and pregnancy in the ROI is crucial to ensure accurate, transparent, and timely information. This approach will enhance the ability to conduct comprehensive research, promote consistency and reproducibility of research findings, and improve public health outcomes.

The implementation of the IHI is currently underway in the ROI. The IHI, which was utilized in the COVAX initiative, has significant potential for streamlining data linkage. Advocating the accelerated rollout of the IHI, akin to the HCN number used for linked datasets available in NI, could further support research and public health efforts. The ability to link individual records from different databases using a unique identifier has many advantages. The use of an IHI enables researchers to combine anonymized administrative datasets to answer research questions without intrusion into patients' lives, particularly if individuals are severely ill. It is an efficient and cost-effective method to conduct population-based research that is feasible during emergency response. In this instance, it would mean that each data source would not need to collect the same data, and each data source could concentrate on collecting quality data in one area (e.g., COVID-19 test) and be linked to another data source (e.g., clinical maternal records with pregnancy outcome data). In relation to research on exposure during pregnancy, the study would not be impeded by an unknown pregnancy status or early pregnancy as the pregnancy status would be obtained from the maternity records, and the gestational age at infection or vaccination can be accurately estimated, which is essential for safety studies.

Access to metadata i,e, detailed information on all variables as well as definitions of these variables and the size of the data in each dataset was limited in ROI. Effectively managing data relies on both clear documentation and an understanding of its scale. A data dictionary is essential for ensuring consistency, clarity, and proper documentation of variables, as understanding the variable definitions, formats, and relationships is crucial. Due to the lack of metadata in ROI, it is not possible to assess the consistency between variables in the ROI datasets. In NI, the variables recording covid-19 infections, vaccinations, types of vaccinations in the NIMATS data will be compared to the infection (Pillar 1, Pillar 2) and vaccination data to assess consistency and reliability. To our knowledge, there have been no studies assessing the rate of infections and vaccination use in pregnant women on the IOI. The NI team have published studies using the NIMATS data ^19, 20^.

Additionally, detailing the size of the data would inform computational requirements, needed storage capacity, and the feasibility of different analytical approaches. Standardized procedures for using anonymized linked data ensure that accessing sensitive data on COVID-19 and pregnancy complies with legal and ethical requirements, further protecting the privacy and rights of the public. Transparent processes promote accountability when data access and usage protocols are upheld. This helps prevent misuse or unauthorized access to pregnancy data and fosters accountability for any breach or misconduct. To further enhance access to large datasets, funding agencies and academic institutions should establish a formal partnership with health services. This collaboration can help navigate the practical steps required, despite legislative changes. A dedicated funding body could facilitate coordination between these groups and promote effective data sharing. This process was developed at NI, where the HBS within the HSC provides data access to researchers.

## Future directions

In the ROI, a national standardized data access process with transparent expected timelines would help to reduce the complexity of accessing COVID-19 and pregnancy data. Further implementation of the IHI system, similar to the HCNs in NI, will enable seamless data linkage across various healthcare sectors. A linked data network can ensure uniform data collection and ease of access for research purposes. An Italian study proposed that a data-driven framework could ensure real-time data updates regarding the risk of COVID-19 and enable the identification of high-risk areas ^21^. Furthermore, as suggested by Maher *et al.*, the formation of data science teams (embedded in organizations such as the National Perinatal Epidemiology Centre) to assist with data management and handling of data requests in ROI would enhance the provision of pregnancy data for secondary use ^16^.

To further enhance the scope and quality of research on COVID-19 and pregnancy, improving data linkages and accessibility across nations is crucial. One key approach is to establish a joint task force between the ROI and NI to facilitate cross-border data sharing.

To reduce the application timelines for accessing data on COVID-19 and pregnancy in the ROI and NI, implementing clear guidelines and support mechanisms for researchers would help expedite data access. This should include detailed information on what is required in an application and what criteria are used for evaluation and approval. Furthermore, support mechanisms such as Frequently Asked Questions (FAQs), dedicated personnel, and help desks that can assist researchers in navigating the application process would be beneficial. Additionally, synthetic data to allow piloting of data cleaning and analysis syntax would allow researchers to progress their research while waiting for data to be made available. Transparent communication channels would keep researchers informed and up-to-date regarding the status of an application and expected timelines to help manage funders’ expectations. Clear and transparent timelines would optimize the process of data access for research on COVID-19 and pregnancy, using secondary data.

Throughout the COVID-19 pandemic, challenges and concerns related to reducing the data entry burden on healthcare workers and protecting patient privacy became key topics of debate. Now referred to as "documentation burden" the extensive administrative tasks healthcare workers must perform, particularly related to patient records, is identified as a significant contributor to healthcare worker burnout ^22^. The demands of the COVID-19 pandemic further exacerbated this burden. Streamlining the documentation process is not without its challenges. As outlined in this paper, the development of a national maternity repository that integrates data from various sources could reduce the data entry burden on healthcare professionals. By streamlining data collection processes, this electronic system would perhaps minimise the duplication of efforts on behalf of hospital staff and enhance the efficiency of data management especially in the ROI where data linkage is currently not possible.

Furthermore, the rapid increase in health data collection throughout the pandemic has raised concern regarding the security and potential misuse of sensitive personal information. While specific studies detailing pregnant women's concerns about data breaches following the COVID-19 pandemic remain limited, the broader issue of health data protection is highly debated. This paper highlights the use of anonymized data as a key strategy to increase willingness to share information by addressing privacy concerns and regulatory requirements (e.g., GDPR). The removal of personally identifiable information would aim to reduce the risk of re-identification, misuse, and ethical concerns, fostering trust among healthcare providers, institutions, and patients. In NI, the anonymized data are made available to researchers in a “safe haven” within the Honest Broker Service, with strict processes to ensure data protection. All researchers must complete the Safe Researcher Training before access will be granted.

## Pandemic preparedness

Timeliness of data access is important in a pandemic situation. Timely access to data is needed not only to contribute to international research efforts but also to quickly assess the impact of the new infection during pregnancy and to assess the safety of the vaccine for pregnant women as it is rolled out, particularly in relation to rarer outcomes such as CA. Pandemic preparedness should include a focus on information systems and how they will provide important information about pregnancy and pregnancy outcomes. Accurate information is needed regarding pregnancy status, most importantly in early pregnancy, and gestational age in relation to exposures, such as infection and vaccination, since the impact of exposure is gestational age-dependent. Precise data regarding the timing of pregnancy was available in NI but were limited in ROI in this study. Given the critical nature of the first trimester in congenital anomaly development, accurate information correlating the timing of both COVID-19 infection and COVID-19 vaccination with gestational stage is essential for identifying potential associations with such anomalies. Inevitably, an unknown early pregnancy may result in a false-negative pregnancy status; however, further administrative delays should be avoided. Emergency responses often neglect to consider the benefits of collecting data for research, with immediate priorities of surge capacity. A recommendation is to record the date of changes to the metadata when they are made to allow for the accurate interpretation of missing or unknown data fields. Rapid data access systems for pandemics should be planned, including explicit access to pregnancy data.

## Conclusion

The process of accessing health data in both the ROI and the NI was rigorous, with many safeguards to protect the privacy of the public’s administrative data and the security of sensitive data. Several challenges hinder the acquisition of anonymized data on COVID-19 infection and vaccination uptake during pregnancy, particularly in the ROI. These include data fragmentation, privacy concerns, incomplete reporting, lack of standardization surrounding data access procedures, and issues related to data quality. In the ROI, delays in obtaining data, even after acquiring ethical approval, led to significant setbacks in research timelines. The absence of clear procedural guidance outlining the steps involved in accessing the requisite data in the ROI further complicates the process. In summary, the contrasting processes between NI and ROI underscore the complexities inherent in accessing data on COVID-19 infection and vaccination rates during pregnancy. To effectively address these challenges, it is essential to focus on improving the research infrastructure, including data-sharing protocols, and simplifying access procedures in the ROI. These steps will promote the development of strong research initiatives and enable informed decision making within public health policies.

## Ethics & consent

This project was approved by the Ethics Committee of Cork Teaching Hospitals for Cork University Maternity Hospital (CUMH): ECM 4 (b) 01/08/2023 & ECM 3 (c) 05/12/2023. Following approval, a research application form was submitted to the Local Information Governance Group.

## Data Availability

No data were analysed for this article. Data sources referenced in this paper are not open access. In the ROI, to initiate the process of accessing CIDR data, a data access request form outlining details of the proposed project must be submitted to CIDR’s National Peer Review Coordinator ( cidrdatarequests@hpsc.ie). To access COVAX data, a COVAX Data Share Request form outlining the proposed project, including insurance details (liability), must be submitted to Integrated Information Services (IIS) ( iis.team@hse.ie), the main data analytics service for the HSE. To access MN-CMS data from Cork University Maternity Hospital (CUMH), ethical approval must be sought by the Clinical Research Ethics Committee of Cork Teaching Hospitals (CREC). In NI, access to COVID-19 infection, COVID-19 vaccination, and NIMATS databases were obtained via the HBS ( https://bso.hscni.net/).
