# Supplementary material for: Data sources on COVID-19 infection and vaccination in pregnancy on the island of Ireland: strengths, weaknesses, and recommendations for future pandemic preparedness
Source: HRB Open Res. 2025 Mar 7;7:77. Originally published 2024 Dec 12. [Version 2] doi: 10.12688/hrbopenres.14011.2 (PMC11920687; doi:10.12688/hrbopenres.14011.2)
Supplement: Peer review history

# Reviewer report: Reviewer response for version 2

**DOI:** 10.21956/hrbopenres.15498.r46332

**Published:** 2025-03-18

**Authors:** Ai Hori

**Affiliations:** 1 University of Tsukuba, Tsukuba, Japan

I have reviewed the author's response to my comments. As a result, I acknowledge that the quality of the paper has improved at each step. Thank you for the opportunity to review this informative paper.

Is the work clearly and accurately presented and does it cite the current literature?

Partly

If applicable, is the statistical analysis and its interpretation appropriate?

Not applicable

Are all the source data underlying the results available to ensure full reproducibility?

Partly

Is the study design appropriate and is the work technically sound?

Partly

Are the conclusions drawn adequately supported by the results?

Partly

Are sufficient details of methods and analysis provided to allow replication by others?

Partly

Reviewer Expertise:

Public health

I confirm that I have read this submission and believe that I have an appropriate level of expertise to confirm that it is of an acceptable scientific standard.

# Reviewer report: Reviewer response for version 2

**DOI:** 10.21956/hrbopenres.15498.r46331

**Published:** 2025-03-12

**Authors:** Qiuyuan Qin

**Affiliations:** 1 University of Rochester Medical Center, Rochester, USA

Thank you for the opportunity to review the manuscript. The authors sufficiently addressed the comments. I have no more comments.

Is the work clearly and accurately presented and does it cite the current literature?

Yes

If applicable, is the statistical analysis and its interpretation appropriate?

Not applicable

Are all the source data underlying the results available to ensure full reproducibility?

Yes

Is the study design appropriate and is the work technically sound?

Yes

Are the conclusions drawn adequately supported by the results?

Yes

Are sufficient details of methods and analysis provided to allow replication by others?

Partly

Reviewer Expertise:

NA

I confirm that I have read this submission and believe that I have an appropriate level of expertise to confirm that it is of an acceptable scientific standard.

# Reviewer report: Reviewer response for version 1

**DOI:** 10.21956/hrbopenres.15377.r44593

**Published:** 2025-01-28

**Authors:** Qiuyuan Qin

**Affiliations:** 1 University of Rochester Medical Center, Rochester, USA

Thank you for the opportunity to review the manuscript. The author described the data sources on COVID-19 infection and vaccination and listed the strength and weakness of the datasets. In general, the author summarized the datasets well. I have a few comments regarding the included variables for the datasets and the use of the datasets.

 Methods:

 The author briefly introduced the data sources and summarize the availability of unique identifications. The included variables and the definitions of the variables, whether the definitions are consistent were not mentioned, especially for some variables of interest, such as covid-19 infections, vaccinations, types of vaccinations, pregnancy timing.

 Results:

 The author summarized each dataset separately and included a strengths and weakness of data sources. It would be better to include more detailed description of the datasets, such as included variables, size of the data and how often the data was updated. In this way, it would provide a clearer guide for researchers about the dataset. In addition, it would also be helpful to include a description of the studies that utilized those datasets.

Is the work clearly and accurately presented and does it cite the current literature?

Yes

If applicable, is the statistical analysis and its interpretation appropriate?

Not applicable

Are all the source data underlying the results available to ensure full reproducibility?

Yes

Is the study design appropriate and is the work technically sound?

Yes

Are the conclusions drawn adequately supported by the results?

Yes

Are sufficient details of methods and analysis provided to allow replication by others?

Partly

Reviewer Expertise:

NA

I confirm that I have read this submission and believe that I have an appropriate level of expertise to confirm that it is of an acceptable scientific standard, however I have significant reservations, as outlined above.

# Response

**Published:** 2025-02-28

**Authors:** Melissa Kelly

**Affiliations:** School of Public Health, University College Cork, Cork, County Cork, Ireland

Dear Qiuyuan Qin,

 We thank you for your detailed summary and helpful review of our manuscript entitled *“Data sources on COVID-19 infection and vaccination in pregnancy on the island of Ireland: strengths, weaknesses, and recommendations for future pandemic preparedness”*.

 Please find below an itemized reply addressing each suggested change in your Reviewer’s Report.

1. *The author briefly introduced the data sources and summarize the availability of unique identifications. The included variables and the definitions of the variables, whether the definitions are consistent were not mentioned, especially for some variables of interest, such as covid-19 infections, vaccinations, types of vaccinations, pregnancy timing.*

Thank you for bringing this to our attention. To address comments made by a previous reviewer, we have now amended Table 2 (see revised manuscript) to include the following detailed headings:

- “Availability or date of data collection, relevant variables included, how often data were updated, who was responsible for data collection, when and where data were collected, average length of data access, application to access, who can access this data, and metadata available.”

The name of Table 2 has also been edited to reflect these changes and is now called:

- “ **Table 2.** Description of data sources on the island of Ireland.”

To address the comment about the size of the datasets, we have added the following information to lines 48-50:

- “There is no information on the size of the datasets in ROI, but the MN_CMS dataset will cover all women and babies in the maternity services in the ROI when the electronic health records are developed.”

Lines 51-52:

- “the national NI maternity system (NIMATS) which records information on approximately 25,000 births per year in NI ….”

Lines 55-56:

- “The Pillar 1, Pillar 2, and vaccination datasets cover the whole population of NI.”

Lines 276-277:

- “Additionally, detailing the size of the data would inform computational requirements, needed storage capacity, and the feasibility of different analytical approaches.”

To address the included variables and definitions of variables within each dataset:

- In NI, all variables are described in the available metadata which can be found via the link in Table 2. In ROI, due to a lack of a data dictionary, we have limited information on included variables and consistency of these variables within the datasets.

We have further addressed this within the manuscript by adding the following text to the Discussion on lines 265-269:

- “Access to metadata i,e,  detailed information on all variables as well as definitions of these variables and the size of the data in each dataset was limited in ROI. Effectively managing data relies on both clear documentation and an understanding of its scale. A data dictionary is essential for ensuring consistency, clarity, and proper documentation of variables, as understanding the variable definitions, formats, and relationships is crucial.”

To address the comment made on whether the definitions of variables of interest are consistent, we have added the following text to the manuscript (lines 269-273):

- “Due to the lack of metadata in ROI, it is not possible to assess the consistency between variables in the ROI datasets. In NI, the variables recording covid-19 infections, vaccinations, types of vaccinations in the NIMATS data will be compared to the infection (Pillar 1, Pillar 2) and vaccination data to assess consistency and reliability.”

Types of vaccines used in ROI and NI throughout the COVID-19 pandemic included Pfizer, Astra Zeneca, Moderna, and Janssen. To address the comment regarding information on types of vaccinations, we have added the following information in the manuscript to the Results section on lines 109-111 for ROI:

- “Vaccines administered in ROI throughout the COVID-19 pandemic included Pfizer, Astra Zeneca, Moderna, Novavax, and Janssen.”

Also, on lines 188-189 for NI:

- “Vaccines administered in NI throughout the COVID-19 pandemic included Pfizer, Astra Zeneca, Moderna, and Novavax.”

To address the lack of detailed information on pregnancy timing in ROI, the following text was added to the Discussion on lines 347-351:

- “Precise data regarding the timing of pregnancy was available in NI but were limited in ROI in this study.  Given the critical nature of the first trimester in congenital anomaly development, accurate information correlating the timing of both COVID-19 infection and COVID-19 vaccination with gestational stage is essential for identifying potential associations with such anomalies.”

 

1. *The author summarized each dataset separately and included a strengths and weakness of data sources. It would be better to include more detailed description of the datasets, such as included variables, size of the data and how often the data was updated. In this way, it would provide a clearer guide for researchers about the dataset. In addition, it would also be helpful to include a description of the studies that utilized those datasets.*

As alluded to above (point 1), there is a lack of detailed information on each of variables collected or size of the data available. We have addressed this within the manuscript by adding the following text to the Discussion on lines 265-269:

- “Access to metadata i,e,  detailed information on all variables as well as definitions of these variables and the size of the data in each dataset was limited in ROI. Effectively managing data relies on both clear documentation and an understanding of its scale. A data dictionary is essential for ensuring consistency, clarity, and proper documentation of variables, as understanding the variable definitions, formats, and relationships is crucial.”

And lines 276-277:

- “Additionally, detailing the size of the data would inform computational requirements, needed storage capacity, and the feasibility of different analytical approaches.”

To address comments on how often/when data were updated, this information was added for each data source to Table 2 under the heading ‘how often data were updated’.

 To address the comment of providing a description of additional studies that have utilised these data sets, we have added the following text to the manuscript on lines 69-71

- “To our knowledge, there have been no studies assessing the rate of infections and vaccination use in pregnant women on the IOI. The NI team have published studies using the NIMATS data.”

The following two studies have been referenced within the text of the manuscript.

- Given J, Casson K, Dolk H and Loane M (2022). Sociodemographic variation in prescriptions dispensed in early pregnancy in Northern Ireland 2010-2016. PLoS ONE 17(8): e0267710. https://doi.org/10.1371/journal. pone.0267710 ; and
- Dolk H, McCullough N, Callaghan S,Casey F, Craig B, Given J, Loane M, et al. (2020) Risk factors for congenital heart disease: The Baby Hearts Study, a population-based case-control study. PLoS ONE 15(2): e0227908. https://doi.org/10.1371/journal.pone.0227908

We hope these amendments address the suggestions raised. Should any further amendments be necessary, we would be happy to address them accordingly.

 Sincerely,

 Melissa Kelly, Joanne Given, Julie Arnott, Helen Dolk, Richard A. Greene, Ali S. Khashan, Seamus Leonard, Mairéad Madigan, Mary T. O’Mahony, Maria Loane, and Gillian M. Maher

# Reviewer report: Reviewer response for version 1

**DOI:** 10.21956/hrbopenres.15377.r44100

**Published:** 2025-01-16

**Authors:** Ai Hori

**Affiliations:** 1 University of Tsukuba, Tsukuba, Japan

This literature provides regional experience about COVID-19 infection and vaccination data collection and access. The information might be meaningful as most countries worldwide also faced problems with data accessibility and data quality (accuracy, reliability, or representativeness) during the pandemic.

 This manuscript will improve by adding more detailed information.

 1. Readers outside the region and the following generation will need to know more detailed data collection and accessibility processes. Please consider adding practical information (When, Who, Where, What, How). For example, who entered the CIDR data (secretary, nurse, or other healthcare professional)? Were everyone in the world allowed to request data access online?

 2. Table 2.

 More detailed headings are needed, not only "strength" or "limitation".

 The detailed heading means; "Average length of the data access approvement", "Availability of date of data collection", or "Available metadata item", etc. The ambiguous term should be avoided in this table. i.e., "time consuming" "may be" or "?" 

 3. Discussion

 Rapid access to data for researchers is essential to improving public health, but in the real world, this cannot be achieved without dialogue between various stakeholders.

 In the discussion section of this paper, it is helpful to explain what debates have taken place during this pandemic so far about reducing the burden on medical workers who enter data and protecting the personal information of those involved, such as pregnant women.

Is the work clearly and accurately presented and does it cite the current literature?

Partly

If applicable, is the statistical analysis and its interpretation appropriate?

Not applicable

Are all the source data underlying the results available to ensure full reproducibility?

Partly

Is the study design appropriate and is the work technically sound?

Partly

Are the conclusions drawn adequately supported by the results?

Partly

Are sufficient details of methods and analysis provided to allow replication by others?

Partly

Reviewer Expertise:

Public health

I confirm that I have read this submission and believe that I have an appropriate level of expertise to confirm that it is of an acceptable scientific standard, however I have significant reservations, as outlined above.

# Response

**Published:** 2025-02-28

**Authors:** Melissa Kelly

**Affiliations:** School of Public Health, University College Cork, Cork, County Cork, Ireland

Dear Ai Hori,

 We thank you for your detailed summary and helpful review of our manuscript entitled *“Data sources on COVID-19 infection and vaccination in pregnancy on the island of Ireland: strengths, weaknesses, and recommendations for future pandemic preparedness”*.

 Please find below an itemized reply addressing each suggested change in your Reviewer’s Report.

1. *Readers outside the region and the following generation will need to know more detailed data collection and accessibility processes. Please consider adding practical information (When, Who, Where, What, How). For example, who entered the CIDR data (secretary, nurse, or other healthcare professional)? Were everyone in the world allowed to request data access online? *

**Response: **Thank you for this suggestion. We have now added practical details regarding who, where and how this data were collected. This has been addressed within the appropriate subheadings of the sources within the Results section as well as Table 2 (see revised manuscript).

 CIDR data collection details on lines 74-77:

- “A multidisciplinary range of healthcare and public health professionals collected CIDR data throughout the COVID-19 pandemic, including public health nurses, doctors, and laboratory staff.”

Addressing who can access CIDR data on lines 80-90:

- “Access to CIDR data is restricted and requires approval from the CIDR Peer Review Group following a thorough application review.”

COVAX data collection details on lines 100-101:

- “A range of healthcare and administrative staff collected COVAX data including vaccination center staff, hospital staff, and pharmacists.”

MN-CMS data collection details on line 127:

- “by hospital staff, including midwives and obstetricians.”

NIMATS data collection details on lines 149-150:

- “NIMATS data are collected by hospital staff, including midwives and obstetricians, during appointments.”

COVID-19 Infection Database (Pillar 1) details on lines 160-162:

- “Testing within pillar 1 is typically conducted using polymerase chain reaction (PCR) and data were collected by hospital staff, including nurses and doctors.”.

COVID-19 Infection Database (Pillar 2) details on lines 166-167:

- “Data were collected by testing center staff, and self-reported data from at home testing kits were uploaded onto the COVID-19 government dashboard.”

COVID-19 Vaccination Database details on lines 186-187:

- “These data were collected by vaccination center staff, hospital staff, and pharmacists.”

To address how often/when data was updated, this information was added for each data source to Table 2 (see revised manuscript) under the heading ‘how often data were updated’.

 To address who was able to access the data, this information was added for each data source to Table 2 under the heading ‘who can access this data’.

1. *More detailed headings are needed, not only "strength" or "limitation". The detailed heading means; "Average length of the data access approval", "Availability of date of data collection", or "Available metadata item", etc. The ambiguous term should be avoided in this table. i.e., "time consuming" "may be" or "?" *

**Response: **We have now amended Table 2 to include the following detailed headings:

- “ *Availability or date of data collection, relevant variables included, how often data were updated, who was responsible for data collection, when and where data were collected, average length of data access, application to access, who can access this data, and metadata available.*”

The name of Table 2 has also been edited to reflect these changes and is now called:

- “ **Table 2.** Description of data sources on the island of Ireland.”

*Rapid access to data for researchers is essential to improving public health, but in the real world, this cannot be achieved without dialogue between various stakeholders. In the discussion section of this paper, it is helpful to explain what debates have taken place during this pandemic so far about reducing the burden on medical workers who enter data and protecting the personal information of those involved, such as pregnant women.*

**Response: **Thank you for this interesting comment. We have added the following paragraph to our discussion beneath the Future Directions sub-heading on lines 316-339:

- “Throughout the COVID-19 pandemic, challenges and concerns related to reducing the data entry burden on healthcare workers and protecting patient privacy became key topics of debate. Now referred to as "documentation burden" the extensive administrative tasks healthcare workers must perform, particularly related to patient records, is identified as a significant contributor to healthcare worker burnout (Rossetti et al. 2021). The demands of the COVID-19 pandemic further exacerbated this burden. Streamlining the documentation process is not without its challenges. As outlined in this paper, the development of a national maternity repository that integrates data from various sources could reduce the data entry burden on healthcare professionals. By streamlining data collection processes, this electronic system would perhaps minimise the duplication of efforts on behalf of hospital staff and enhance the efficiency of data management especially in the ROI where data linkage is currently not possible.

Furthermore, the rapid increase in health data collection throughout the pandemic has raised concern regarding the security and potential misuse of sensitive personal information.  While specific studies detailing pregnant women's concerns about data breaches following the COVID-19 pandemic remain limited, the broader issue of health data protection is highly debated. This paper highlights the use of anonymized data as a key strategy to increase willingness to share information by addressing privacy concerns and regulatory requirements (e.g., GDPR).  The removal of personally identifiable information would aim to reduce the risk of re-identification, misuse, and ethical concerns, fostering trust among healthcare providers, institutions, and patients.  In NI, the anonymized data are made available to researchers in a “safe haven” within the Honest Broker Service, with strict processes to ensure data protection.  All researchers must complete the Safe Researcher Training before access will be granted.”

 We hope these amendments address the suggestions raised. Should any further amendments be necessary, we would be happy to address them accordingly.

 Sincerely,

 Melissa Kelly, Joanne Given, Julie Arnott, Helen Dolk, Richard A. Greene, Ali S. Khashan, Seamus Leonard, Mairéad Madigan, Mary T. O’Mahony, Maria Loane, and Gillian M. Maher
